# Evidence That Field Muskmelon (*Cucumis melo* L. var. *agrestis* Naud.) Fruits Are Solids of Revolution

**DOI:** 10.3390/plants12244186

**Published:** 2023-12-18

**Authors:** Ke He, Cang Hui, Weihao Yao, Jinfeng Wang, Lin Wang, Qiying Li, Peijian Shi

**Affiliations:** 1 Architectural Design and Research Institute, Shenzhen University, #3688 Nanhai Avenue, Shenzhen 518000, China; hkqq5@szu.edu.cn; 2 Bamboo Research Institute, College of Ecology and Environment, Nanjing Forestry University, #159 Longpan Road, Nanjing 210037, China; whyao@njfu.edu.cn (W.Y.); jfwang@njfu.edu.cn (J.W.); lwang@njfu.edu.cn (L.W.); lqyin@njfu.edu.cn (Q.L.); 3 Centre for Invasion Biology, Department of Mathematical Sciences, Stellenbosch University, Stellenbosch 7602, South Africa; chui@sun.ac.za; 4 Mathematical and Physical Biosciences, African Institute for Mathematical Sciences, Cape Town 7945, South Africa

**Keywords:** drought-tolerant plant, explicit Preston equation, field muskmelon, fruits shape, measurement error, vine

## Abstract

In nature, the fruit shapes of many plants resemble avian eggs, a form extensively studied as solids of revolution. Despite this, the hypothesis that egg-shaped fruits are themselves solids of revolution remains unvalidated. To address this, 751 *Cucumis melo* L. var. *agrestis* Naud. fruits were photographed, and the two-dimensional (2D) boundary coordinates of each fruit profile were digitized. Then, the explicit Preston equation (EPE), a universal egg-shape model, was used to fit the 2D boundary coordinates to obtain the estimates of the EPE’s parameters of each fruit. Under the hypothesis that egg-shaped fruits are solids of revolution, the fruit volumes were estimated using the solid of revolution formula based on the estimated EPE’s parameters. To test whether the fruits are solids of revolution, the fruit volumes were measured by using a graduated cylinder and compared with the estimated volumes using the solid of revolution formula. The EPE was demonstrated to be valid in describing the 2D profiles of *C. melo* var. *agrestis* fruits. There was a significant correlation between the measured fruit volumes using the graduated cylinder and the estimated fruit volumes using the solid of revolution formula based on the estimated EPE’s parameters. Acknowledging potential measurement errors, particularly fruit fuzz causing air bubbles during volume measurements, we recognize slight deviations between measured volumes and estimated values. Despite this, our findings strongly suggest that *C. melo* var. *agrestis* fruits are solids of revolution. This study contributes insights into the evolutionary aspects of fruit geometries in plants with egg-shaped fruits and introduces a practical tool for non-destructively calculating fruit volume and surface area based on photographed 2D fruit profiles.

## 1. Introduction

Fruits, exhibiting diverse forms, stand out as highly specialized plant organs within angiosperms, playing a pivotal role in orchestrating the maturation and dispersal of seeds. They mark the culmination of the reproductive cycle in angiosperms [1,2]. Beyond their botanical significance, fruits serve as a crucial food source for numerous frugivorous animals. Fleshy fruits, in particular, attract frugivores, leading them to consume the fruit and subsequently transport the seeds away from the parent plant, mitigating intraspecific competition [3,4]. This symbiotic interaction yields benefits for both parties involved. Frugivorous animals derive nutrition and energy from the fleshy pulp of the fruit [5,6], while plants gain from the seed dispersal facilitated by these animals, fostering gene flow and expanding their distribution range [7,8]. The dynamics of the interaction between fruits and frugivorous animals are significantly influenced by fruit traits, including shape, size, color, scent, and texture [9,10,11,12]. The choice of frugivores becomes a critical factor in this interplay. There is a growing interest in studying these traits, with a particular emphasis on fruit size and shape [13]. These investigations contribute substantially to our comprehension of the mechanisms propelling fruit evolution.

The size of fruit often stands as a critical factor for frugivores in their selection among conspecific fruits [14,15]. This factor extends its influence to the feeding behavior of birds, determining whether they opt to swallow the entire fruit or peck at fragments of its flesh [16]. Notably, larger fruits might face rejection during a single feeding session as birds, despite their physical capability, may be unwilling to ingest larger seeds and bear the burden of flying with them [16,17]. Similarly, the shape of the fruit can significantly impact its likelihood of being consumed by frugivores, owing to specific scaling relationships between fruit size and length [9]. Beyond ecological considerations, fruit shape holds substantial importance in both agronomy and commerce [18]. In the commercial sphere, grapes with an elongated and symmetrical shape command high attraction, prompting ongoing hybridization efforts in grape breeding to accentuate this distinctive elongated trait [19]. Fruit shape also exerts a considerable influence on various commercial aspects, including pricing, market packaging, and quality grade classification.

While the shape of fruits holds significance in various contexts, previous studies have generally lacked a rigorous mathematical quantification of fruit shape, with only a handful achieving such precision [13,20]. In the realm of natural forms, numerous fruit shapes bear resemblance to solids of revolution, yet the extent to which they adhere to this geometric concept remains uncertain. A solid of revolution is a geometric construct typically formed by rotating a curve around a straight line, known as the axis of revolution. Three-dimensional geometry earns the designation of a solid of revolution when its two-dimensional (2D) profile displays bilateral symmetry along its mid-line (coinciding with the axis of revolution), and the 2D projected area remains invariant regardless of the rotation angle about its mid-line. Essentially, if a fruit conforms to being a solid of revolution, its shape can be accurately generated by revolving its 2D profile by π [13].

**Figure 1:**
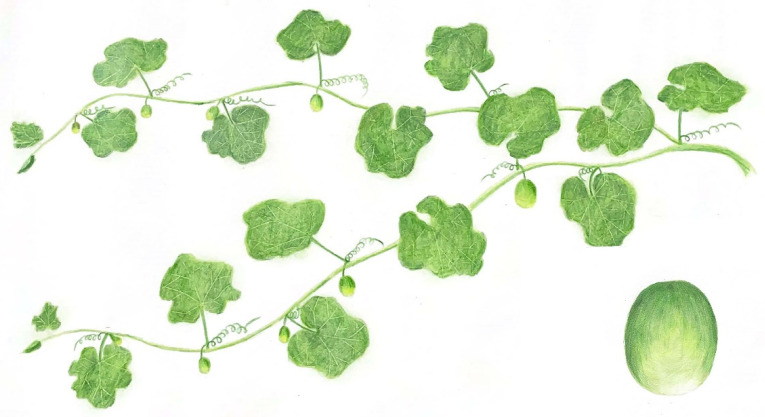
Freehand drawing of the aboveground part and a fruit of *C. melo* var. *agrestis*.

The Cucurbitaceae family has ca. 1000 species, and some have been cultivated into important crops and ornamental plants [21]. Some Cucurbitaceae species have typical egg-shaped fruits, e.g., *Citrullus lanatus* (Thunberg) Matsumura and Nakai, *Cucumis melo* L., and *Cucumis melo* L. var. *agrestis* Naud. The objective of this study was to delineate the shape of field muskmelon (*C. melo* var. *agrestis*) fruits, a member of the Cucurbitaceae family and an annual vine native to regions in Africa, China, and India, which is a drought-tolerant vine [22]. Illustrated in Figure 1 are the aboveground section and a representative fruit of *C. melo* var. *agrestis*. This wild melon boasts a subtly sweet flavor and holds considerable nutritional value. Beyond its culinary appeal, it also possesses noteworthy medicinal properties, including anti-inflammatory, analgesic, antioxidant, and hypoglycemic attributes [23]. Moreover, the seeds of field muskmelon are high in fat content, rendering them suitable for the production of edible oils. This quality positions *C. melo* var. *agrestis* as a potentially significant oilseed crop [24]. There are two reasons that we selected this species as the study material: (i) its fruit shape resembles avian eggs, and (ii) its fruit size is smaller than that of other Cucurbitaceae plants (e.g., *C. lanatus* and *C. melo*), which makes it easier to measure the fruit volume.

The configuration of *C. melo* var. *agrestis* fruits shares similarities with that of avian eggs, a phenomenon extensively investigated and modeled in various studies (e.g., Refs. [25,26,27,28,29,30]). In a recent development, Shi et al. [31,32] employed the explicit Preston equation, denoted as EPE henceforth, to portray the 2D profiles of 2221 eggs from six avian species. They then compared the predicted volumes, generated using a solid of revolution formula based on EPE’s estimated parameters, with empirically measured volumes from two of the six avian species. This comparison affirmed the accuracy of EPE in characterizing avian egg profiles and lent support to the hypothesis that avian eggs can be considered to be solids of revolution. Motivated by the insights gained from the study of avian egg shapes, our research aims to explore the effectiveness of EPE in describing the 2D geometries of *C. melo* var. *agrestis* fruits. We seek to determine whether the fruit geometry aligns with the characteristics of a solid of revolution.

## 2. Materials and Methods

### 2.1. Fruit Sampling

**Figure 2:**
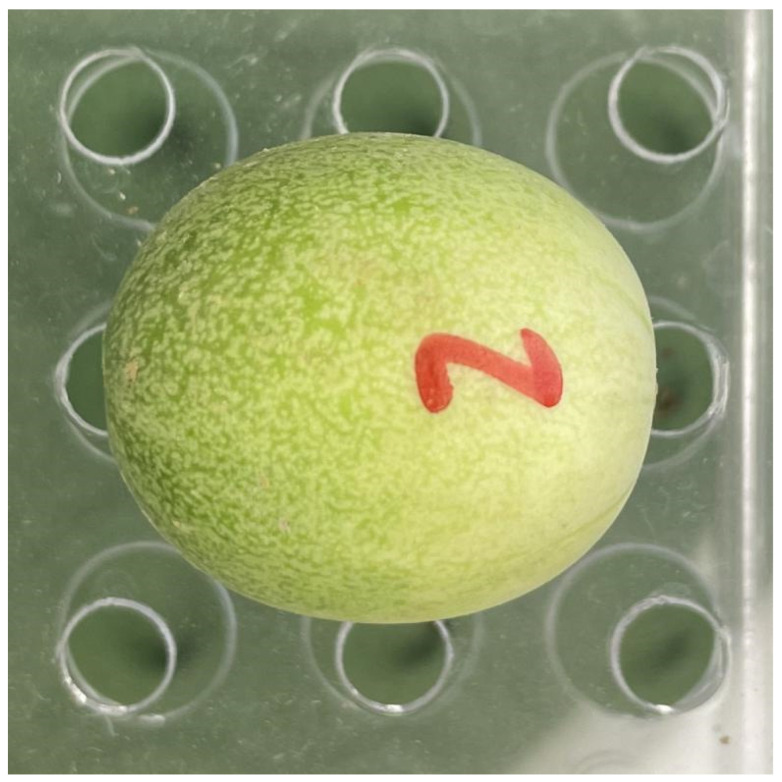
A representative *C. melo* var. *agrestis* fruit image.

A total of 751 mature and undamaged fruits were sampled from 31 *C. melo* var. *agrestis* plants that were naturally growing in Changgou Town (117°44′26″ N, 33°29′14″ E), Sixian, Anhui Province, China, in early August 2023. To obtain the aboveground portion of each individual plant, the vine at ground level was severed. Subsequently, the collected fruits were placed in storage boxes and transported to the laboratory within an hour. Figure 2 provides a representative image of the fruit.

### 2.2. Photographing and Data Acquisition

A horizontal smartphone (iPhone 12, Zhengzhou, China) was fastened securely by an adjustable tabletop phone mount to capture images of *C. melo* var. *agrestis* fruits. To ensure that the fruits were stably positioned horizontally and that the lens was focused on the fruit’s center, a test tube rack was directly positioned beneath the camera. Additionally, we measured the length of each fruit using a vernier caliper (0–150 mm, Shanghai Accurate Measuring Tools Co., Ltd., Shanghai, China; measurement accuracy: 0.02 mm) to calibrate any deviation in image size from its actual dimensions.

The photographs were converted into black-and-white images, cropped into a rectangular shape, and saved as bitmap images at 600 dpi using Adobe Photoshop CS2 (version 9.0; Adobe, San Jose, CA, USA). The planar coordinates of each fruit profile were extracted using a function developed by [33,34] based on Matlab (version ≥ 2009a; MathWorks, Natick, MA, USA). The “adjdata” function in the “biogeom” package (version 1.3.5) [35] based on R (version 4.2.0) [36] was employed to digitize the profile of each *C. melo* var. *agrestis* fruit.

### 2.3. Modeling and Data Fitting

The mathematical expression of the explicit Preston equation (EPE) [25,31,32] is

(1) $$y=\pm b\sqrt{1-{\left(\frac{x}{a}\right)}^{2}}\left(1+c_{1}\left(\frac{x}{a}\right)+c_{2}{\left(\frac{x}{a}\right)}^{2}+c_{3}{\left(\frac{x}{a}\right)}^{3}\right)$$

where *x* and *y* are the horizontal and vertical coordinates in the Cartesian plane, representing an arbitrary point on a 2D fruit profile; *a* and *b* represent half the length and approximately half the maximum width of the fruit, respectively. Parameters *a*, *b*, *c*_1_, *c*_2_ and *c*_3_ are to be estimated. The positive and negative signs on the right-hand side of Equation (1) represent the upper and lower parts of the fruit profile, with its midline aligned along the *x*-axis.

On the assumption that *C. melo* var. *agrestis* fruits are solids of revolution, their volume (*V*) and surface area (*S*) can be calculated using Equations (2) and (3) [37]:

(2) $$V=\pi \int_{-a}^{a}y^{2}dx$$

and

(3) $$S=2\pi \int_{-a}^{a}y\sqrt{1+{\left(\frac{dy}{dx}\right)}^{2}}dx$$

where d*y*/d*x* represents the derivative of *y* with respect to *x*; and other parameters remain consistent with those of Equation (1). An analytical solution of the volume formula based on Equation (1) can be obtained using Equation (2) [31]:

(4) $$V=\frac{4}{315}\pi ab^{2}\left(105+21c_{1}^{2}+42c_{2}+9c_{2}^{2}+18c_{1}c_{3}+5c_{3}^{2}\right)$$

The “fitEPE” function in the “biogeom” package (version 1.3.5) [35] based on R (version 4.2.0) [36] was used to fit the data points of the fruit profile to estimate the values of *a*, *b*, *c*_1_, *c*_2_ and *c*_3_. The minimization of the residual sum of squares (RSS) between the observed and predicted *y*-values on the 2D profile of *C. melo* var. *agrestis* fruits was achieved using the Nelder–Mead optimization method [38]. The adjusted root-mean-square error (RMSE_adj_), which represents the proportion of the mean deviation in *y* values to half of the fruit’s maximum width, was used to determine the goodness-of-fit between the observed and predicted data [31,32]:

(5) $${\mathrm{RMSE}}_{\mathrm{adj}}=\frac{\sqrt{{\sum_{i=1}^{N}\left(y_{i}-{\hat{y}}_{i}\right)}^{2}/N}}{W/2}$$

where the subscript *i* represents the *i*th data point of the fruit profile; *N* represents the number of data points of the fruit profile; and *W* represents the maximum width of a fruit. As a rule of thumb, RMSE_adj_ < 0.05 reflects a satisfying goodness-of-fit for the curve fitting.

### 2.4. Testing the Solid of Revolution Hypothesis

Ensuring that the calculated numerical values of volume (*V*) and surface area (*S*), obtained through Equations (2) and (3), closely align with or show negligible differences compared to the observed values of *V* and *S* allows the inference that the hypothesis of a solid of revolution holds true. Accurately measuring *S* for the fruit poses challenges, while obtaining observed values of *V* is relatively straightforward using a graduated cylinder. If the fruit’s profile aligns well with Equation (1), and the theoretically calculated value of *V* using Equation (4) is identical or nearly identical to the observed *V*, this further supports the solid of revolution hypothesis.

To conduct the test, the EPE was employed to fit the 2D profile of the fruits. Subsequently, we used the solid of revolution formula, Equation (4), based on the estimated parameters of EPE, to predict the volumes of the 751 fruits. The observed volumes of the fruits were measured by individually submerging them in a 100 mL cylinder with a 3 cm diameter, recording the numerical changes in volume scale before and after submersion. Subsequently, we compared the predicted volume with the observed volume. 

### 2.5. Statistical Analysis

Reduced major axis protocols [39,40] were used to fit the observed and predicted volumes of *C. melo* var. *agrestis* fruits. The bootstrap percentile method [41,42] was used to calculate the 95% confidence interval (CI) of the slope of the regression line with the intercept equal to zero. If the CI of the slope includes unity, it can indicate that the predicted volumes are not significantly different from the observed volumes, which means that the fruits can be treated as solids of revolution. All calculations and figures were carried out using R (version 4.2.0) [36].

## 3. Results

**Figure 3:**
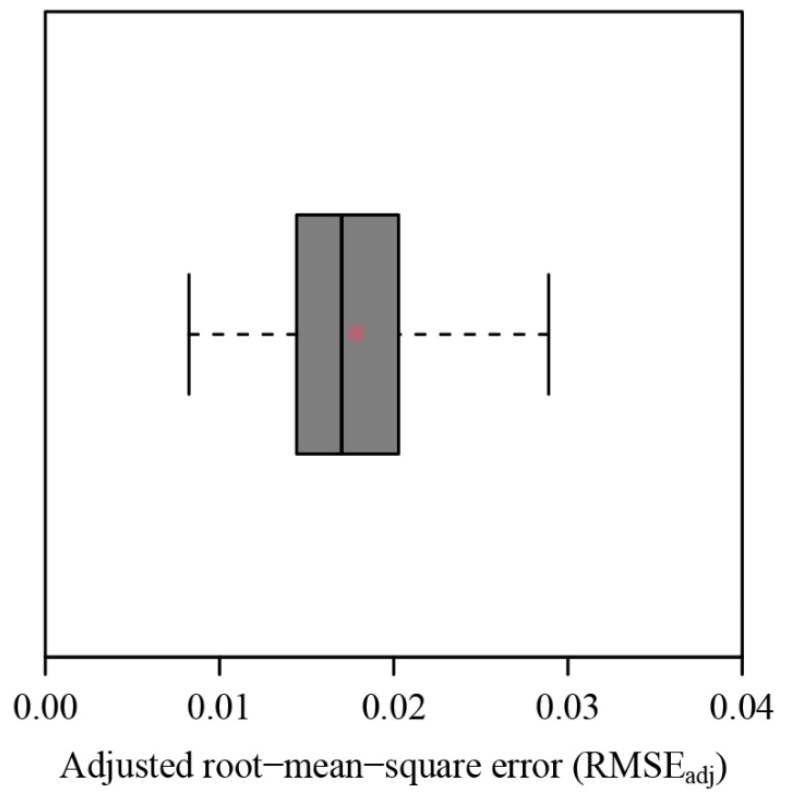
Box-and-whisker plot of the adjusted root-mean-square errors (RMSE_adj_) calculated using Equation (5). The vertical solid line in the box represents the median, and the asterisk within the box represents the mean.

**Figure 4:**
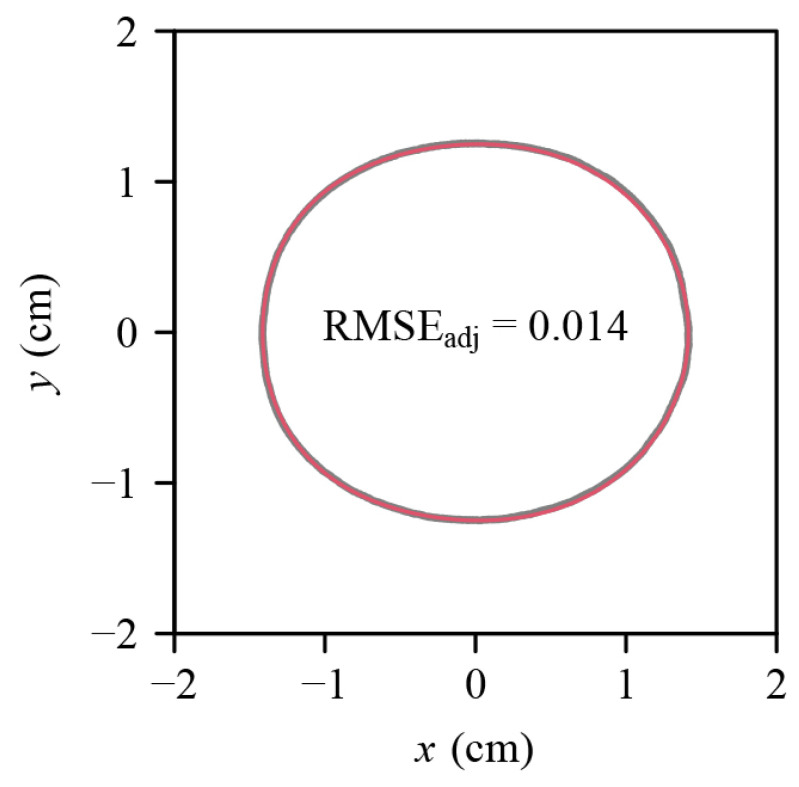
Fitted result of a *C. melo* var. *agrestis* fruit example (corresponding to Figure 2) using the explicit Preston equation (i.e., Equation (1)). The gray curve represents the observed geometry, and the red curve represents the predicted geometry. RMSE_adj_ is the adjusted root-mean-square error between the observed and predicted values.

All the adjusted RMSE values for the 751 *C. melo* var. *agrestis* fruits are below 0.05, ranging from 0.0083 to 0.0380. This verified the validity of the explicit Preston equation (EPE) for describing the 2D profiles of the fruits (Figure 3). Figure 4 provides the fitted result for a fruit example in which gray and red lines represent the observed and predicted fruit profiles. The fitted results for the 751 fruits were tabulated in the online Supplementary Table S1.

**Figure 5:**
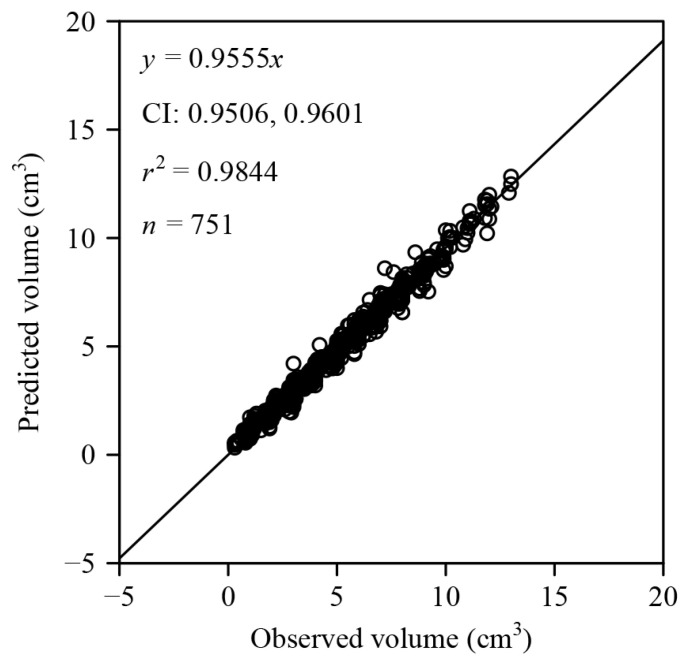
Linear fit with zero-intercept for the observed and predicted volumes of *C. melo* var. *agrestis* fruits. Here, *n* is the sample size, i.e., the number of fruits; *r^2^* represents the coefficient of determination that reflects the linear relationship between the two interdependent variables; *x* of an open circle is the observed volume using the graduated cylinder; *y* of an open circle is the predicted volume calculated using Equation (2) based on the explicit Preston equation; CI provides the 95% confidence interval of the slope.

The 95% CI for the slope of the observed and predicted fruit volumes were 0.9506 and 0.9601 (Figure 5). As the 95% CI does not include unity, there is a significant difference between the observed and predicted volumes. However, as the coefficient of determination (i.e., *r*^2^) is equal to 0.9844, there is a good linear relationship between the two sets of fruit volumes. This implies that the hypothesis of the solid of revolution holds, although it could have been disturbed due to experimental errors.

## 4. Discussion

A comparison was made between the volumes predicted by the explicit Preston equation (EPE) and those measured using a graduated cylinder for 751 *C. melo* var. *agrestis* fruits. Notably, a significant disparity in volume (*V*) emerged between the two methods, casting doubt on the validity of the EPE and, consequently, challenging the hypothesis that *C. melo* var. *agrestis* fruits can be regarded as solids of revolution. Further discussion of these aspects will be undertaken in the subsequent subsections.

### 4.1. Validity of the Explicit Preston Equation for Describing Fruits

The EPE represents a more explicit mathematical expression compared to Preston’s original formulation from 1953 and demonstrates a robust fit to the 2D profile of *C. melo* var. *agrestis* fruits. Preston’s equation, initially designed for modeling avian egg shapes, notably excels in accurately depicting the 2D profile of avian eggs, particularly pyriform eggs. While alternative mathematical equations exist for describing avian egg profiles [28], the universal egg-shape equation introduced by [29] and validated using data from nine avian species stands out [30]. Additionally, Shi et al. [30] found that a simplified version of the polar coordinate equation proposed by Gielis [43] yielded a better fit for egg-shape data from the same nine bird species. However, among the available egg-shape equations, the EPE appears to be the optimal choice, providing the best goodness of fit [32].

There are primarily two methods for estimating the parameters of the EPE or its simplified versions: the multiple linear regression and nonlinear optimization methods [32]. The multiple linear regression method assumes that the longest axis on the egg’s profile represents the mid-line, implying perfect bilateral symmetry. However, this assumption often does not hold true due to photographic or placement errors. Therefore, it is crucial to consider bilateral asymmetry along the mid-line of the egg’s profile. The nonlinear optimization method is employed to estimate the parameters of the EPE, taking into account the bilateral asymmetry of digitized egg profiles by automatically searching for the optimal mid-line and providing a good fit.

It is evident that the photographed 2D profiles of *C. melo* var. *agrestis* fruits cannot be perfectly bilaterally symmetric due to photographic and placement errors. In this study, the method of Shi et al. [31] was used to capture images of *C. melo* var. *agrestis* fruits. Measurement errors during fruit photography, such as the misalignment of the smartphone camera’s center and the fruit’s center not forming a vertical line to the horizontal surface, alongside the non-horizontal placement of the fruit’s midline, may contribute to deviations from perfect bilateral symmetry. Additionally, the softness of *C. melo* var. *agrestis* fruits may result in slight deformations, further challenging perfect bilateral symmetry. In such instances, employing a nonlinear optimization method that considers an angle deviation between the fruit’s midline and the *x*-axis [32] is advantageous over the traditional multiple linear regression method, as it can address the imperfect bilateral symmetry of the 2D profiles of *C. melo* fruits.

### 4.2. Are C. melo var. agrestis Fruits Solids of Revolution?

In the zero-intercept linear regression analysis between observed and predicted fruit volumes, the 95% confidence interval of the slope did not include unity, indicating a statistically significant difference (Figure 5). However, the coefficient of determination exceeded 0.98, signifying a strong linear relationship between the two sets of fruit volumes. This result hints at the potential presence of measurement errors during the experimental process.

The surfaces of *C. melo* var. *agrestis* fruits are covered with numerous small fuzzes. Submerging a fruit in a graduated cylinder generates many small air bubbles due to these fuzzes. The volume of these small bubbles becomes included as part of the fruit volume, leading to an overestimate of the observed volume using the graduated cylinder. Some *C. melo* var. *agrestis* fruits float in the water, while others remain exposed above the water surface. To ensure complete submersion during volume measurement, we used a thin wire to press the fruits down. However, this process may also cause a part of the wire to be submerged, resulting in an overestimation of the observed volume.

**Figure 6:**
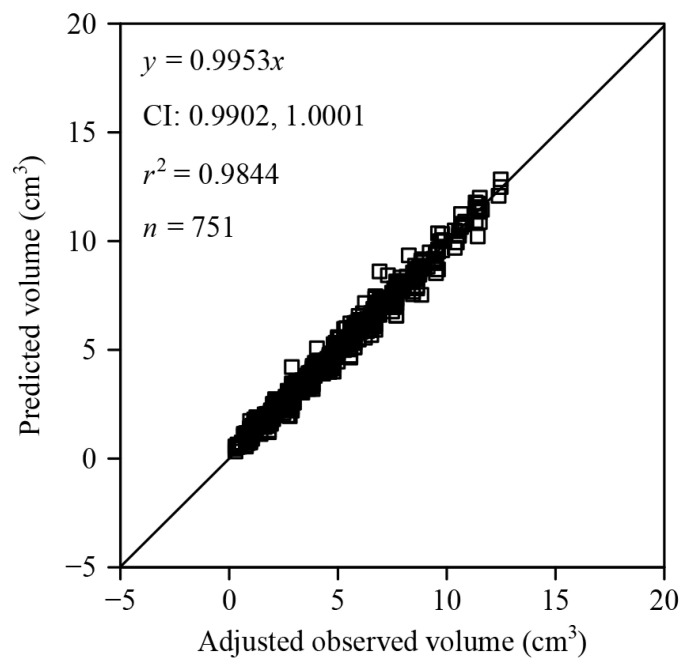
Linear fit with zero-intercept for the adjusted observed and predicted volumes of *C. melo* var. *agrestis* fruits. Here, *n* is the sample size, i.e., the number of fruits; *r*^2^ represents the coefficient of determination that reflects the linear relationship between the two interdependent variables; *x* of an open square is the adjusted observed volume (i.e., the actual observed volume multiplied by 0.96) using the graduated cylinder; *y* of an open square is the predicted volume calculated using Equation (2) based on the explicit Preston equation; CI provides the 95% confidence interval of the slope.

To address this, we multiplied all observed fruit volumes by a range of candidate ratios from 0.600 to 0.995 at intervals of 0.005. We found that the ratio of 0.96 minimized the sum of squared residuals between the predicted volumes and the adjusted observed volumes. Using a linear equation with the intercept equal to zero to fit the predicted volumes and the adjusted observed volumes, the confidence interval of the slope included unity (Figure 6), suggesting no significant difference between the predicted volumes and the adjusted observed volumes. This finding implies that *C. melo* var. *agrestis* fruits likely exhibit characteristics of solids of revolution.

## 5. Conclusions

This study affirms the applicability of the explicit Preston equation (EPE) in characterizing the 2D profiles of *C. melo* var. *agrestis* fruits, as indicated by adjusted root-mean-square errors consistently below 0.05. Despite the 95% confidence interval of the slope for the linear regression (with the intercept equal to zero) between predicted volumes, using the solid of revolution formula based on EPE, and the measured volumes using the graduated cylinder, not encompassing unity, a robust correlation between the two volume datasets was observed. Considering the impact of bubbles introduced by fruit fuzzes during volume measurements using the graduated cylinder, there is strong justification to assert that *C. melo* var. *agrestis* fruits are highly likely to be solids of revolution. This work not only validates the presence of solids of revolution in nature, particularly among egg-shaped fruits, but also suggests that the estimated parameters of the EPE hold potential for comparing variations in fruit shape within and across species, offering insights into the evolutionary aspects of fruit morphology.

## Data Availability

The data can be found in the online Supplementary Table S1.

## Supplementary Materials

The following supporting information can be downloaded at https://www.mdpi.com/article/10.3390/plants12244186/s1, Table S1: The estimated parameters of the explicit Present equation and fruit size parameters of the 751 *Cucumis melo* var. *agrestis* fruits.
